# Medical Society of the County of Erie

**Published:** 1910-04

**Authors:** Franklin C. Gram


### CORRESPONDENCE.

### Medical Society of the County of Erie

**Celebration of Dr. Jacobi's Birthday.—Members of the Medical Society of the County of Erie requested to contribute one dollar each.**

*Editor Buffalo Medical Journal*:

Sir—The following circular letter, signed by Dr. Charles Jewett, President, Dr. J. Riddle Goffe, Secretary, and Drs. A. T. Bristow and Joseph D. Bryant, Committee of Arrangement for the celebration of Dr. Jacobi's birthday, has just been received, and at the direction of President Grover W. Wende a copy is mailed to each member:

At the last meeting of the Medical Society of the State of New York the House of Delegates took the following action which was unanimously adopted:

*Resolved*, That a committee consisting of the Presidents of the Medical Society of the State of New York, and of the recent New York State Medical Association be and hereby is appointed to consider and carry into effect a celebration to be given in honor of Dr. Abraham Jacobi on his 80th birthday.

In order that this celebration may be available to all members of the society and oppressive to none, it is decided to give Dr. Jacobi a reception at the New York Academy of Medicine on Friday evening, May 6, 1910, and to present him with a substantial testimonial of appreciation.

Believing that each member of the society will be delighted to coöperate in this purpose, thus testifying to his appreciation of, and affection for a noble character, it is proposed that each member be requested as an earnest of his willingness and desire, to subscribe $1.00 to the cause and to be present on the occasion. Therefore you will kindly take the matter up at once with the members of your county society and report promptly to the committee of arrangements, as it must have, at an early date, a knowledge of the fund which will be at its disposal.

Personal invitations will be issued in due time.

President Wende requests all those who desire to contribute, in accordance with this announcement, to communicate either with him or Dr. Stockton, or send their contributions direct to Dr. Albert T. Lytle, Treasurer, who will make due acknowledgment and forward the amount to the Committee of Arrangement.

FRANKLIN C. GRAM,

Secretary.

March 3, 1910.

---

"Why, what are all these bottles for? Have you been taking medicine?" she asked, coming upon them suddenly.

"Yes," they answered, grasping at straws.

